# Informed Choices and Early Dementia Risk Disclosure in Home‐Based Assessments in the PREDICTOM Study: A Public Involvement Approach

**DOI:** 10.1002/alz70861_108833

**Published:** 2025-12-23

**Authors:** Sarah Campill, Dianne Gove, Ana Diaz, Anna‐Katharine Brem, Zunera Khan, Ellie Pickering, Dag Aarsland, Jean Georges, The PREDICTOM consortium

**Affiliations:** ^1^ Alzheimer Europe, Luxembourg, Senningerberg Luxembourg; ^2^ Alzheimer Europe, Senningerberg, Luxembourg Luxembourg; ^3^ University Hospital of Old Age Psychiatry, University of Bern, Bern Switzerland; ^4^ King’s College London, London UK; ^5^ Institute of Psychiatry, Psychology and Neuroscience, King's College London, London UK; ^6^ University of Exeter, Exeter UK; ^7^ Stavanger University Hospital, Stavanger Norway; ^8^ Centre for Healthy Brain Ageing, IoPPN, King’s College London, London UK

## Abstract

**Background:**

The rapid progress in detecting dementia risk through emerging digital biomarkers and biofluid markers, alongside the development of disease modifying treatments (DMTs), underscores the importance of this evolving field. The PREDICTOM study leverages technological advances to develop an AI‐driven platform for home‐based early diagnostic assessment. Here, we explore mitigating the potential emotional impact of risk disclosure to participants post at‐home screening phase based on previous work and publication by Alzheimer Europe on ethics and risk disclosure.

**Method:**

In parallel with the development of the multicentre study, in which n=4000 individuals over the age of 50 with increased risk of developing AD are recruited across 7 European sites, a Public Ambassador Group (PAG) of people from the general public with and without lived experience (*n* =7) from five European countries was set up to share their views, concerns and feedback with the project partners. PAG members were consulted to discuss participants' needs and expectations regarding post‐at‐home screening (level 1) feedback and risk disclosure for participants who are included as well as excluded from level 2. Insights from these consultations informed the development of a resource for informed decision‐making, helping participants understand the emotional impact of risk disclosure. This work was followed up by the development of a decision‐making tool based on a bottom‐up approach involving the PAG, the European Dementia Carers Working Group (EDCWG) and the European Working Group of People With Dementia (EWGPWD).

**Result:**

Interim results from the PI work for PREDICTOM highlight the need for a tool to help participants make informed choices about risk disclosure, avoiding distress due to a lack of understanding. This is particularly relevant for early dementia detection studies, where participants might underestimate their risk.

**Conclusion:**

The PREDICTOM study will offer a pioneering resource for research participants in early dementia risk detection studies, co‐created with public input. Key components and the preliminary version of the decision‐making tool will be presented at the AAIC.

